# Effect of Different Water Cooling Treatments on Changes in Rectal and Surface Body Temperature in Leisure Horses after Medium-Intensity Effort

**DOI:** 10.3390/ani12040525

**Published:** 2022-02-21

**Authors:** Iwona Janczarek, Anna Wiśniewska, Ewelina Tkaczyk, Elżbieta Wnuk-Pawlak, Beata Kaczmarek, Marta Liss-Szczepanek, Witold Kędzierski

**Affiliations:** 1 Department of Horse Breeding and Use, Faculty of Animal Sciences and Bioeconomy, University of Life Sciences in Lublin, 20-950 Lublin, Poland; iwona.janczarek@up.lublin.pl (I.J.); ewelina.tkaczyk@up.lublin.pl (E.T.); elzbieta.wnuk@up.lublin.pl (E.W.-P.); khiuk@up.lublin.pl (M.L.-S.); 2 Department and Clinic of Animal Internal Diseases, Faculty of Veterinary Medicine, University of Life Sciences in Lublin, ul. Akademicka 13, 20-950 Lublin, Poland; beatakaczmar1@gmail.com; 3 Department of Biochemistry, Faculty of Veterinary Medicine, University of Life Sciences in Lublin, 20-950 Lublin, Poland; witold.kedzierski@up.lublin.pl

**Keywords:** carrying off heat, internal temperature, thermography, thermoregulation

## Abstract

**Simple Summary:**

The study was performed in order to answer the question: should horses be cooled after not very intensive exercise? Thus, the efficiency of four different water cooling methods was studied in horses after medium-intensity effort for leisure horses under moderate air temperature. The water cooling methods used included spraying with cold water: (1) lower body parts, i.e., lower parts of legs; (2) upper body parts, i.e., back of the head and flanks; and (3) both lower and upper body parts. The control group was not treated with water. Water cooling was applied to 19 warmblood geldings immediately after the end of exercise and 10 and 20 min later. The internal and body surface temperatures were registered and analyzed. The water cooling methods used led to a decrease in rectal and body surface temperature. Applying cold water on lower body parts was only effective and can be recommended for practical use under the described conditions.

**Abstract:**

Cooling a horse after intensive exercise under hot conditions is commonly recommended. The study aimed to analyze changes in the rectal and surface temperature of the horses subjected to various water cooling treatments. This followed medium-intensity exercise performed by leisure horses under moderate air temperature. The experiment involved a control group without water application, and three variants of water cooling applied to 19 warmblood geldings after medium-intensity effort. Cooling of lower, upper, and lower and upper body parts was performed. In each variant, the rectal and body surface temperatures were measured five times: before; immediately after; and 10, 20, and 30 min after effort. Using water cooling under the studied conditions did not influence a post-exercise decrease in the rectal temperature. The decrease in body surface temperature depended on the used variant of cooling down the horse. Cooling the limbs by pouring water several times changed the surface body temperature from 34.2 ± 0.37 °C to 32.0 ± 0.32 °C and was more efficient than the repeated application of cool water on both the upper and lower body parts, leading to a temperature change from 34.6 ± 0.26 °C to 33.2 ± 0.36 °C. Thus, the application of cold water on the limbs only is sufficient for cooling the horse after medium-intensity exercise under moderate air temperature (about 24 °C).

## 1. Introduction

The body temperature of warm-blooded organisms is controlled by a thermoregulation center located in the preoptic area and the anterior region of the hypothalamus that regulates heat production in biochemical processes in a living organism [1,2]. Generally, land mammals lose excess heat mainly through the skin by long-term heat radiation, convection, evaporation, and conduction, as well as by the respiratory tract as a result of evaporation [3,4]. The body can radiate heat in several ways, and it depends on the surface area-to-volume ratio of the body part [5,6]. In horses, due to the high value of this ratio in the limbs, lack of muscles, and low vascular supply in these parts of the body, it is a place of intense heat exchange. The temperature of these body parts is correlated with the temperature of the environment. Moreover, at low air temperatures, local vascular valves direct blood from the limbs upwards to minimalize heat loss through convection, while the situation is reversed at high temperatures [7].

Due to the high surface area-to-weight ratio, horses are susceptible to overheating, i.e., hyperthermia [8]. Thermal stress may occur and cause general weakness, consciousness disorders, accelerated pulse, breathing disorders, exhausted horse syndrome, synchronous flutter diaphragm (SDF), thermal shock, exercise rhabdomyolysis, and anhidrosis, and, in extreme cases, thermal stress leads to death [9,10]. Hyperthermia is initiated by both external (high air temperature and humidity) and internal (physical effort, cardiovascular diseases, obesity) factors or by a combination of these factors [11]. Thus, various cold applications have been tested to cool sport horses after exercise [12,13]. Moreover, pouring water on the legs activates blood circulation, thus reducing the risk of swelling. Cool water seems to be the most effective method of cooling horses after exercise; however, the effectiveness of this method was studied under hot, humid conditions (30 °C and relative humidity of 80%) [13]. This method involves pouring cold water on a horse or applying ice to selected parts of its body. The authors’ own observations indicate that riders, especially amateurs, overuse the application of cold water even after not very intensive exercise or under moderate ambient temperature (20–24 °C), in which the temperature is still in the range of thermo-neutral zone for recreation horses, and the horse can independently cope with dissipating heat produced in the body [11]. Although the occurrence of hyperthermia in horses after recreational riding is very unlikely, inexperienced riders in fear of the condition of their horses often unnecessarily and excessively cool them. Water cooling leads to an immediate (but short-term) restriction of the vessels, causing an increase in the internal temperature in the initial phase [14]. This is followed by long-term vasodilation, which initiates heat dissipation from the body. Therefore, both internal and external temperature should be considered when assessing the effectiveness of heat dissipation from the body. Horses with a significantly elevated temperature should be cooled immediately by using a hose spraying cold water on the body, especially on the neck, shoulders, back, and legs [11]. These actions can generally result in improved thermal comfort. Horses with increased rectal temperature raised to no more than 38.5 °C usually respond well to simple methods, such as resting in a shady place, drinking a small amount of water, or cooling limbs with cold water to restore homeostasis [11,13,15]. Cooling the horse’s limbs is especially beneficial for tendon homeostasis [16,17]. Generally, all of the methods mentioned above proved to be effective compared to the application of tepid water (31 °C) or passive cooling of horses [18].

Thus, cooling a horse after intensive exercise in hot, humid conditions is beneficial. Nowadays, a large proportion of riders are amateurs who use horses for recreational riding only. Therefore, it is worth considering whether water cooling should be applied after a low- or medium-intensity effort for leisure horses (see further down) and, if so, to what extent. This paper poses the hypothesis that effecting water cooling in leisure horses after medium-intensity efforts can be achieved through spraying with water certain body parts only and applied over certain periods. On the basis of this hypothesis, this study aimed to analyze changes in the rectal and surface temperature of leisure horses after various water cooling treatments following medium-intensity effort.

## 2. Materials and Methods

### 2.1. Animals

Animal care and the experimental procedures were in accordance with the European Committee Regulations on Protection of Experimental Animals and were approved by the Local Ethics Review Committee for Animal Experiments (no. 27/2016).

Experiments in four repetitions were carried out on 19 warmblood geldings. The geldings were Anglo-Arabian half-breed horses weighing 523 ± 28.7 kg and aged from 10 to 15 years. Each of the 19 horses took part in four repetitions of the experiment. All of the experimental horses were regularly trimmed and not shoed. The animals were clinically healthy and did not exhibit any behavioral anomalies. The horses had summer hair (not clipped) and were brushed vigorously for 30 min every morning for 10 days prior to the experiment and during the study. All of the horses had been kept for at least 12 months in the same stable in 3.5 × 3.5 m boxes with openwork partitions, which enabled mutual eye contact. There was a manger in the corner of each box, an automatic drinking bowl and a hay basket. The horses were fed fodder consisting of 3 kg of meadow hay and 1.5 kg of oats, three times daily (at 6.00 a.m., 12.00 a.m., and 6.00 p.m.), as well as mineral and vitamin supplements in doses recommended by the manufacturer, once a day (Platinum horse mineral, Soymax, Lublin, Poland). Fresh water and salt blocks were still available. The floor in the boxes was bedded with wheat-rye straw once daily and was cleared every morning. The stable was ventilated naturally on a round-the-clock basis, which helped to maintain the temperature at 20–21 °C during the day and 15–17 °C at night. Every morning, the horses were used for recreational riding for approximately 60 min in an outdoor, unshaded riding arena, and subsequently walked for approximately 20–30 min. Additionally, on hot days (above 30 °C), after exercise, the horses were cooled down with tepid water by pouring it on their limbs and the skin on the back of their heads, neck, and back. This was a normal procedure in this stable. After these procedures, they were left on a partly shaded pasture for at least three hours.

### 2.2. Experiment

The experiment was conducted in the summer (between 23 June and 12 August). In order for the conditions of the experiment to be optimized, exercise was started early in the morning on hot days and later in cooler weather. Moreover, horses were exercised inside a 17 × 55 m indoor arena (riding hall) with a professional riding ground. The conditions inside the arena were estimated using the psychrometric method. The air temperature was 23.6 ± 3.4 °C, atmospheric pressure: 996.6 ± 17.0 hPa, air speed: 0 ± 0.0 m/s, and the air relative humidity was 62.4 ± 18.0%.

Each of the 19 horses was exercised four times during the experiment, according to the scheme presented in Table 1. In the experiment, the work of a horse without a rider was considered because the influence of a rider on a horse may change the physiological parameters of the horse, for example, the rider’s weight influenced the post-exercise superficial horse body temperature [19]. The horses performed lunge-work (see further down), saddled but without a rider, which was intended to be equivalent to medium-intensity effort of studied horses in comparison to their routine work with a rider [20]. A bridle with a snaffle bit, a Hanoverian noseband, a chambon, and a lunge line were used. No horse boots were used.

The scheme of the lunge-work (total time on the left and right side) was as follows: 5 min of walking, 20 min of trotting, 5 min of walking, 5 min of cantering, 5 min of trotting, 5 min of cantering, 10 min of trotting, and 5 min of walking. The horses switch back and forth between working to the left and right every 2.5 min. The mean speed of walking, trotting, and cantering was calculated on the basis of the lap time measured with a hand stopwatch and the circumference of the circle on which the horses moved while working on the lunge. It amounted to 77.4 ± 9.6 m/min, 173 ± 11.4 m/min, and 223 ± 13.8 m/min, respectively. During each repetition of the experiment, four horses (1–4) worked the first day, the next four horses (5–8) worked on the second day, the next four horses (9–12) worked on the third day, another four horses (13–16) worked on the fourth day, and the last three horses (17–19) worked on the fifth day. The horses were randomly assigned to consecutive days of the first repetition of the experiment. Each horse was used in the study four times, every 10 days. In subsequent repetitions, the assignment of the horses to particular days remained the same as in the first repetition.

The work of the horses was organized in 10 to 15 min intervals (horse-by-horse) in order to ensure sufficient time intervals for taking measurements. In each repetition, the order of leading the horses to the arena was changed according to a fixed scheme so that each horse in each repetition was led in a different order.

**Figure 1:**
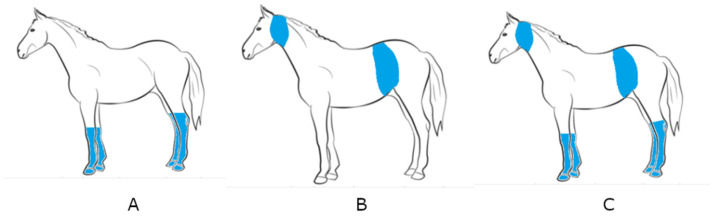
Scope of the horse body cooling. (**A**): lower body parts, (**B**): upper body parts, (**C**): lower and upper body parts.

Immediately after each repeated exercise, different variants of treatments were applied. The experimental variants were as follows: (1) control variant—without water cooling (CON), (2) lower body parts cooling variant (Low), (3) upper body parts cooling variant (Up), (4) lower and upper body parts cooling variant (Low + Up) (Table 2, Figure 1).

In the CON variant, horses were only walked in a shady area. Walking is the simplest and most basic way to restore a horse’s physiological parameters after exercise and is commonly used in practice [12]. Walking lasted 30 min, and measurements were conducted just before walking in 10 min and 20 min of walking (during short rest made for measurements, lasting 2 min) and 30 min after the end of exercise (end of walking). Cooling the legs is commonly used in practice, whereas the back of the head and croup, point of hip, and flank areas were chosen to be cooled in this study because these parts of the body were covered most with sweat in the studied horses, i.e., more than the neck, whirl, or rump.

Water cooling sessions lasted 30 s, and such a session was applied to each body part of the left and right side of the body in a given variant in each repetition, both after post-effort examination (time “0”, i.e., immediately after the post-effort temperature measurements) and after 10 min and 20 min. Water cooling on the lower body parts was conducted with a hose and a soaking sponge on the upper body parts. The temperature of the used water was tested daily at the beginning and end of the experiment and was 17.0 ± 0.2 °C.

### 2.3. Study Methodology

The horse rectal and surface body temperature measurements were conducted to assess heat removal effectiveness. Temperature-taking in the rectum was a routine procedure in the stable under study and the horses were accustomed to the procedure. It was measured with a veterinary thermometer Veterinär—Thermometer SC 12 (measurement time: 30 s). The body surface temperature was the average temperature of the measurements taken from five regions of interest (ROIs) on the left side of the horse’s body: ROI1—head and neck (the vertical cut-off line at the shoulder joint), ROI2—trunk (vertical cut-off lines at the shoulder joint and the tubercle of the iliac crest, the horizontal cut-off line at the line of the sternum), ROI3—croup (the vertical cut-off line at the tubercle of the iliac crest, the horizontal cut-off line at the stifle joint), ROI4—foreleg (the horizontal cut-off line at the height of sternum), ROI5—hind leg (the horizontal cut-off line at the stifle joint). These areas were combined into the entire left side surface of the horse’s body, on the basis of which the average surface temperature was determined. Only the left side of the horse’s body was taken into account in the measurement because previous studies have shown that the surface temperatures of the right and left sides of the body of a healthy horse do not show significant differences [3,21].

The body surface temperature was measured with a Thermal Imagers Ti9 FLUKE thermal imaging camera (a non-cooled microbolometric matrix operating in the focal plane, resolution 120/160 pixels, IR range 7.5 μm to 14 μm) positioned 250 cm from the horse on a 100 cm-high tripod. The thermographic images (actions associated with taking the image: 60 s) were taken following the applicable procedure: the horse stood still in a dark, closed room at a constant temperature to minimize the effect of atmospheric conditions on the experiment results. In addition, since it was necessary to conduct thermographic examinations, the horses did not have any wraps, blankets or other equipment that could have affected the results [14]. In the next step, the data from the camera were uploaded to a computer and analyzed in SmartView 4.1. software. An analysis was performed of the mean surface temperature of the entire horse: left side lateral view.

All temperature measurements were performed: at rest; before effort (At rest); immediately after the end of effort, i.e., before the first water cooling session (Post-exercise); 10 min after the first water cooling session (10 min recovery); 20 min after the end of exercise, i.e., 10 min after the second water cooling session (20 min recovery); and 30 min after the end of effort, i.e., 10 min after the third water cooling session (30 min recovery). Between the subsequent cooling repetitions, horses rested by walking in the shade. The same measurement routine was performed for the control variant and each studied variant of water cooling (Table 3).

### 2.4. Statistical Methods

The obtained data were tested for normality of distribution using the Shapiro–Wilk method. Normal distribution was confirmed, and an analysis of variance was performed with SAS (Statistical Analysis System, version 9.4, SAS Institute, Cary, NC, USA) using the Mixed model for repeated measurements, including the random effect of the horse and the fixed effects of two factors: the variant of cooling the horse and the temperature measurement stage. The analyzed variants of cooling the horse were as follows: CON, Low, Up, and Low + Up. The analyzed temperature measurement stages were as follows: (1)—At rest, (2)—Post-exercise, (3)—10 min recovery, (4)—20 min recovery, and (5)—30 min recovery. The interactions between main effects were also tested. The results are presented as the least-square means (LSM) with standard errors (SE). The differences between levels of analyzed effects were tested by the post hoc multiple comparison for LSM using Duncan’s test. The level of probability was assumed as 95% (*p* < 0.05).

## 3. Results

No significant differences were recorded in the resting or post-exercise rectal temperature between the studied cooling variants (*p* < 0.05). The post-exercise rectal temperature was significantly higher than the resting temperature in each study variant (Table 4). A significant drop in the rectal temperature in subsequent recovery stages was observed only in the Up variant and the Low + Up variant (Table 4). The rectal temperature recorded in the 30 min recovery stage in the Low + Up variant was significantly lower than that measured in the other variants of horse cooling (Table 4).

No significant differences were noted for the resting and post-exercise average surface temperature between studied cooling variants (*p* < 0.05). Conversely, the differences within one variant were observed in all cases. In each studied variant, the average post-exercise surface temperature was higher than that measured at rest (Table 5).

The average surface temperature during the 10 min recovery stage did not differ in comparison with the post-exercise stage following the application of CON, Low, and Up variants, whereas it significantly decreased in the Low + Up variant (Table 5). In the 30 min recovery stage, the mean surface temperature in the CON variant remained significantly higher than at rest, whereas in each cooling variant the superficial temperature in the 30 min recovery stage did not differ in comparison to its resting value. Moreover, in the 30 min recovery stage, the surface temperature in the CON and Low + Up variants remained significantly higher than in the Low and Up variants (Table 5).

## 4. Discussion

In all experimental cooling variants, both rectal and surface temperatures measured at rest remained at a similar level. The level of resting temperatures is not surprising and is determined by the horse’s warm-bloodedness [2]. As Baptiste [21] reported, warm-blooded organisms strive to maintain a constant inner body temperature, which is needed to maintain the proper function of internal organs, especially the brain. This phenomenon is one of the determinants of a horse’s clinical health [13].

It was also found that the rectal and surface temperatures measured after exercise (post-exercise) did not significantly differ between studied groups. The absence of differences in the post-exercise temperatures (Table 4 and Table 5) is probably caused by similar external conditions throughout the experiment and the intensity of effort, which—according to McCutcheon and Geor [22]—restricts individual post-effort reactions. This means that each horse during the experiment reacted to performed exercise with a rise in internal and superficial temperatures within a similar range. Thus, the day of the experiment and/or the assignment of the horse to a particular cooling variant did not affect the results in the post-exercise stage. However, as expected, both rectal and surface temperatures after exercise were considerably higher than those measured at rest. When evaluating the temperatures in the post-exercise stage, it should be underlined that their values cannot be recognized as causing hyperthermia [8]. An increase in the rectal temperature was reported in horses not only after an intensive effort in hot, humid conditions [12,13] but also in response to medium-intensity exercise [23] or exercise performed in a cool environment [24].

It is generally known that during the post-effort recovery, the rectal temperature should decrease gradually, which is the determinant of the correct course of the thermoregulation process [24]. However, such a correlation was not found in the CON, where horses were not subjected to cooling with water but only to walking in the shade. An elevated rectal temperature during 30 min post-exercise recovery was also reported by Wallsten et al. [24]. The rectal temperature also did not drop in the Low variant. On the basis of the obtained results, we could recommend this type of partial cooling as a standard therapy that can be repeated even in short time intervals. According to other researchers, this method seems safe for health [25], relaxes the tendons and joints after effort [26], and shows a general relaxing effect [27,28]. Moreover, it should be underlined that in case of repeated cooling of the upper body parts, the rectal temperature dropped in the 30 min recovery stage to its resting value. A similar effect was noted in other studies in response to intensive water cooling [13,23,29]. It is therefore worth considering whether the application of local cooling would not cause an excessive decrease in body temperature. In a study by Takahashi et al. [12], the rectal temperature remained elevated for 30 min after exercise, despite pouring cold water on the horse. In fact, the cited experiment was performed in hot and highly humid conditions, which limited evaporation.

On the basis of the surface temperature during the initial 10 min of recovery, we found that differences in the heat dissipation process, which are connected with the experimental cooling variant, were not significant at the 10 min recovery stage. Again, it should be noted that the lack of evident changes in the surface temperature in the first minutes of recovery may have resulted from the not very intensive effort. Therefore, the findings of this study can be regarded as consistent with those cited and in line with the results of the authors’ internal temperature experiments [19]. Thus, this above confirms that after effort that does not cause evident hyperthermia, the standard process of thermoregulation, which aims at heat dissipation, is only discretely initiated by the body. Marlin et al. [13] are of a similar opinion.

The results obtained during the whole post-exercise period indicated that using variant Low and Up significantly decreased the surface body temperature in comparison with the control group and the Low + Up variant. Kang et al. [30] also stated that water application generally has more cooling effect on superficial tissues temperature than rectal temperature. In particular, using the Low variant of cooling resulted in a significant decrease in body surface temperature. Using the Up variant had a similar effect. However, using the Low + Up variant resulted in a less efficient drop of surface temperature than the Low variant. Spraying upper body parts with cold water probably led to the restriction of the blood vessels, causing inhibition in heat dissipation from the body [14].

Therefore, it can be stated that water cooling applied on lower body parts after medium-intensity effort for leisure horses, as studied in the experiment, is sufficient to cool the horse. However, water cooling applied to both the upper and lower body parts seems to be ineffective. This suggestion may be supported by the results published by Monk [31]. This author indicates the necessity of multiple cooling with water of even the entire animal body, but only when the internal temperature strongly increases, i.e., it is almost 2.0–2.5 °C higher than the temperature recorded in the current experiment.

The present study takes into account two parameters: rectal and superficial body temperature. Both parameters increased in response to exercise and decreased or tended to decrease during the restitution period. Similar relationships between superficial and rectal temperature were reported in other studies on exercised horses [12,19,30].

To sum up, the body of the horse does not respond with surface temperature changes to a single post-effort water cooling. Although a single cooling session such as that performed in the experiment can be recognized as typically hygienic or relaxing, it cannot be suggested that it will affect thermoregulation. Thus, if both rider and horse are accustomed to washing the horse after riding, such treatment can be used, although it is unrelated to the cooling of the horse. To efficiently cool the horse after exercise, it is enough to pour cold water several times on the lower body parts only. It was found that after multiple cooling sessions applied on both the lower and upper body parts, a surface temperature drop is less effective. Thus, using repeated water cooling sessions applied on both the upper and lower body parts is not recommended after medium- or low-intensity effort for leisure horses.

Although the course of body temperature changes in the studied horses was analyzed in detail, the study is not without limitations. The workload of horses was evaluated on the basis of the increase in rectal temperature only, while other exercise parameters, such as heart rate and blood lactic acid concentration, were not registered. Thus, the term medium-intensity effort only applies to leisure horses and not to sport or racehorses.

## 5. Conclusions

An analysis of the post-exercise rectal and surface temperature indicates that spraying a horse with water once does not cool it down. For recreational horses, the application of cool water on the horse’s limbs three times after moderate effort seems to be sufficient to cool the horse down. Since the repeated application of cool water on the horse’s upper and lower body parts after medium-intensity work seems to be less effective than the application of cool water on limbs, it should not be recommended after medium- or low-intensity effort for leisure horses.

## Figures and Tables

**Table 1:** General scheme of the experimental procedure performed on 19 horses to test various water cooling treatments and air temperature inside the arena during the study periods (°C).

Experiment Day	Horse Numbers Randomly Assigned in the Experiment	Experimental Cooling Variant	Abbreviation	Air Temperature Inside the Arena during Horse Exercises (°C)
1	1–4	Control group	CON	17.5–22.3
2	5–8			21.6–24.4
3	9–12			24.0–29.9
4	13–16			23.3–22.8
5	17–19			23.1–23.9
6–15		Pause		
16	1–4	Lower body parts water cooling	Low	24.5–30.1
17	5–8			14.6–21.5
18	9–12			23.1–26.2
19	13–16			19.8–24.0
20	17–19			21.5–25.2
21–30		Pause		
31	1–4	Upper body parts water cooling	Up	24.6–30.3
32	5–8			25.0–25.6
33	9–12			16.9–22.3
34	13–16			24.8–23.1
35	17–19			20.4–27.4
36–45		Pause		
46	1–4	Lower and upper body parts water cooling	Low + Up	23.6–28.1
47	5–8			25.0–26.7
48	9–12			19.9–25.3
49	13–16			18.2–22.4
50	17–19			23.6–28.3

Air temperature inside the arena during horse exercises—measurements taken at the beginning and at the end of the horse’s lunging work.

**Table 2:** Study cooling variants after exercise—four sessions.

Cooling Variants		Actions Performed	Duration of Rest (min)	Timing of Application of Water Cooling (min)		
				First	Second	Third
CON	Control (*n* = 19)	Walking in the shade, without water cooling	30	-	-	-
Low	Lower body parts cooling (*n* = 19)	Water cooling of the lower limb parts, including the knee, hock, and fetlock joint, and then walking—repeated three times	30	0	10	20
Up	Upper body parts cooling (*n* = 19)	Water cooling of the upper body parts: back of the head and croup, point of hip and flanks area, and then walking—repeated three times	30	0	10	20
Low + Up	Lower and upper body parts cooling (*n* = 19)	Water cooling of the lower limb parts and upper body parts, and then walking—repeated three times	30	0	10	20

**Table 3:** Experimental procedures during each studied cooling variant.

Measurement Stage	The Studied Cooling Variant	Actions Performed
At rest	all	measuring the internal and superficial temperatures
-	all	lunge work
Post-exercise	all	the post-effort temperature measurement
-	CON variant	walking in the shade
	Low, Up, and Low + Up variants	first application of water cooling, and then walking in the shade up to 10 min
10 min recovery	all	the initial recovery temperature measurement
-	CON variant	walking in the shade
	Low, Up, and Low + Up variants	second application of water cooling, and then walking in the shade
20 min recovery	all	the second recovery temperature measurement
-	CON variant	walking in the shade
	Low, Up, and Low + Up variants	third application of water cooling, and then walking in the shade
30 min recovery	all	the third recovery temperature measurement

**Table 4:** Mean rectal temperatures (°C) measured at rest, immediately after exercise and following repeated stages of cooling in studied horses (LSM ± SE).

Variant/The Measurement Stage	At Rest	Post-Exercise	10 min Recovery	20 min Recovery	30 min Recovery	RMSE	*p*
CON (control)	37.2 ± 0.07 x	38.1 ± 0.10 y	38.0 ± 0.08 y	37.8 ± 0.08 y	37.7 ± 0.09 axy	0.09	0.02
Low (lower body parts cooling)	37.4 ± 0.07 x	38.2 ± 0.09 y	38.1 ± 0.07 xy	37.9 ± 0.07 xy	37.7 ± 0.08 axy	0.08	0.04
Up (upper body parts cooling)	37.5 ± 0.08 x	38.0 ± 0.11 y	38.1 ± 0.09 y	37.8 ± 0.09 xy	37.6 ± 0.09 ax	0.10	0.04
Low + Up (lower and upper body parts cooling)	37.3 ± 0.10 x	38.1 ± 0.12 y	37.9 ± 0.13 y	37.8 ± 0.11 y	37.2 ± 0.10 bx	0.12	0.03
RMSE	0.09	0.11	0.12	0.10	0.10	-	-
*p*	0.22	0.49	0.43	0.67	0.02	-	-

Means marked with similar letters (x, y: in rows; a, b: in columns) do not differ significantly at *p* ≤ 0.05, RMSE—root mean square error.

**Table 5:** Mean surface temperatures (°C) measured at rest, immediately after exercise, and following repeated stages of cooling in the studied horses (LSM ± SE).

Variant/Measurement Stage	At Rest	Post-Exercise	10 min Recovery	20 min Recovery	30 min Recovery	RMSE	*p*
CON (control)	31.8 ± 0.38 x	34.1 ± 0.33 y	34.1 ± 0.33 y	33.5 ± 0.29 y	33.4 ± 0.29 ay	0.37	0.01
Low (lower body parts cooling)	32.4 ± 0.55 x	34.2 ± 0.37 z	33.5 ± 0.35 yz	33.2 ± 0.24 y	32.0 ± 0.32 bx	0.51	0.00
Up (upper body parts cooling)	32.3 ± 0.37 x	34.1 ± 0.29 z	34.0 ± 0.28 z	33.1 ± 0.26 y	32.0 ± 0.23 bx	0.35	0.00
Low + Up (lower and upper body parts cooling)	32.5 ± 0.34 x	34.6 ± 0.26 z	33.6 ± 0.38 y	33.2 ± 0.24 xy	33.2 ± 0.36 axy	0.33	0.00
RMSE	0.49	0.35	0.33	0.28	0.34	-	-
*p*	0.08	0.89	0.34	0.23	0.03	-	-

Means marked with similar letters (x, y, z: in rows; a, b: in columns) do not differ significantly at *p* ≤ 0.05, RMSE—root mean square error.

## Data Availability

The data presented in this study are available on request from the corresponding author.
